# Problems with interpretation of transient hyperemic response ratio (THRR)

**DOI:** 10.1186/s13089-018-0095-2

**Published:** 2018-06-07

**Authors:** Achyut Sharma, Diptesh Aryal

**Affiliations:** Nepal Mediciti Hospital, Lalitpur, Nepal

We read with interest an article by Al-Jehani et al. [1] published recently in your reputed journal. The authors have made a meritorious effort at highlighting the usefulness of a simple yet effective test ‘transient hyperemic response (THR)’ in the prediction of delayed ischemic neurologic deficit (DIND) in subarachnoid hemorrhages (SAH). It is undisputable that an accurately performed and analyzed test of transcranial Doppler (TCD) will help us explicate intricate details about the cerebral pathophysiology. But, whether the same can be said about THR is still a matter of debate especially when considering the interdependence of various neurophysiological parameters. We think that the following points, in reference to the study by Al-Jehani, are worth considering.

Firstly, any ultrasound procedure is liable to operator interpretation and THR is not different [2]. Although, the authors have explicitly mentioned that a single operator performed all the tests, the intra-operator variability in the test hasn’t been mentioned and most probably not possible considering that this is a retrospective data review. For the same reason, the generalizability of the data and replicability of the tests are not sufficiently bolstered. A similar study by Lam et al. [3] has similar limitation in addition to being underpowered with small sample size. However, we do not mean to enfeeble the collective importance of such studies. Secondly, although the authors have well documented individual patient characteristics including the Hunt and Hess grade, the flow velocities in the cerebral arteries are better defined in terms of age group and gender, for example velocities are considered to be higher in females than males between 20 and 60 years [4] whereas the difference is more subtle in the older age group. Thus, the results would have been more valid and credible if they were to be analyzed taking into consideration the physiological variables.

Next, the authors mentioned about repeating the tests every 48 h and made a prediction about DIND based on the initial THR findings. The necessity and more importantly usefulness of a repeated measure THR is not clearly mentioned in the text. Are we assuming that a test that was negative to begin with will always remain negative during the course of the illness? Probably not. The results would have been more credible if the results of the subsequent THR were mentioned. We rather believe that a daily performed test and a subsequent change in flow velocities provide more accurate information rather than an absolute single value [5, 6]. Similarly, when THR is used to predict the onset of vasospasm, the other factors affecting cerebral autoregulation cannot at all be forgotten. The point-of-time mean arterial pressure, etCO_2_ level, vessel anatomy, intracranial pressure (ICP), collateral flow pattern, and hematocrit are just some of the important factors which might alter the interpretation of the THR value [7]. We cannot negate the fact that cerebral autoregulation is active between certain MAP value and completely disregarding this while interpreting the results of THR is not justified. We believe that the results of THR need to be considered taking into account all of these important parameters for this and any future studies to make the result more informative and accurate.

Further, the authors have included a significant number of patients where the aneurysm was in the posterior circulation (Pcom and basilar), but the differences in the interpretation of values have not been mentioned specifically. The flow velocities in the posterior circulations are lower than the middle cerebral circulation. Thus, the same value should not be used for comparing the flow in both the circulations [4]. We would like to, therefore, reiterate the fact that the interpretation of findings between these two circulations need to be made differently also being aware of the fact that posterior vessels are difficult to insonate. Even when considering the velocities in the middle cerebral artery (MCA), we have to be cognizant that flow only in the proximal segment (M1) of MCA is better delineated in TCD and the spasm in the distal segment (M2) is often interpreted indirectly [8].

We like to conclude that although the prediction of DIND using THR is a simple, novel, and reproducible point-of-care test, the interpretation of result is complicated by the effect of multiple factors. Completely disregarding these factors not only invalidates the data, but also makes the whole effort futile. Also, TCD that is repeated and compared to previous will yield a better result. Having said this, we reiterate that TCD and THR certainly have potential to match the usefulness and reliability of cerebral angiography although they cannot completely replace the latter. Further studies comparing these techniques and considering all the potential variables will certainly give us valuable information.

## Abbreviations

DIND: delayed ischemic neurologic deficit
etCO_2_: end tidal CO_2_ concentration
ICP: intracranial pressure
MAP: mean arterial pressure
MCA: middle cerebral artery
Pcom: posterior communicating
SAH: subarachnoid hemorrhage
TCD: transcranial Doppler
THR: transient hyperemic response
THRR: transient hyperemic response ratio
